# Label-free detection and identification of single bacteria via terahertz near-field imaging

**DOI:** 10.3389/fmicb.2023.1195448

**Published:** 2023-06-02

**Authors:** Jie Wang, Liang Peng, Dongxue Han, Teng Zheng, Tianying Chang, Hong-Liang Cui

**Affiliations:** ^1^School of Information and Electrical Engineering, Hangzhou City University, Hangzhou, China; ^2^Shenzhen Institute of Advanced Technology, Chinese Academy of Sciences, Shenzhen, China

**Keywords:** single bacteria, THz near-field image, label-free, noninvasive, identify

## Abstract

In recent years, terahertz (THz) imaging has attracted much attention because of its ability to obtain physical and chemical information in a label-free, noninvasive and nonionizing manner. However, the low spatial resolution of traditional THz imaging systems and the weak dielectric response of biological samples hinder the application of this technology in the biomedical field. In this paper, we report a new THz near-field imaging method for a single bacteria, through the coupling effect of nanoscale radius of probe and platinum gold substrate, which greatly enhances THz near-field signal of biological samples. A THz super-resolution image of bacteria has been successfully obtained by strictly controlling the relevant test parameters such as tip parameters and driving amplitude. By analyzing and processing the THz spectral image, the morphology and inner structure of bacteria have been observed. The method has been used to detect and identify *Escherichia coli* represented by Gram-negative bacteria and *Staphylococcus aureus* represented by Gram-positive bacteria. This application provides a new label-free, noninvasive and nonionizing testing protocol for the detection of single bacteria.

## 1. Introduction

Humans are exposed to bacterial pathogens all the time, which are present not only in infected patients, but also in soil, water, wildlife, domestic animals, and food. With the increase of bacterial infection and drug resistance developed over time of the most infectious diseases caused by pathogenic bacteria, we are facing a major public health problem in the world. It is reported that more than 2.8 million people die from bacterial infections every year (Karbelkar and Furst, 2020). A complete and detailed description of the structure and morphology of different bacterial pathogens plays an important role in many biomedical studies related to the diagnosis and treatment of bacterial infections. The determination of bacterial morphology and spectral information can provide structural and biochemical characteristics of the bacteria, which can be used to distinguish pathogenic from harmless symbiotic bacteria. However, due to resolution limitations, individual bacteria cannot be studied in detail using traditional microscopy techniques (Lucidi et al., 2014). For example, the lateral resolution that can be achieved by using conventional microscopy techniques based on laser excitation is limited by the phenomenon of light diffraction, reaching only half the wavelength of the excitation light (Gahlmann and Moerner, 2014). Therefore, higher resolution is needed to describe the basic structure of bacteria at the subcellular level. Optical nanotechnology based on superresolved fluorescence overcomes the diffraction barrier and can provide typical resolution of 30–100 nm (Rust et al., 2006). However, their lack of chemical sensitivity and reliance on tailor-made fluorescent probes limit their wide applicability. For biological samples, the contrast agents used in fluorescence technology can seriously affect the morphology, metabolism and movement of organisms, and may lead to cytotoxicity and phototoxicity (Cosentino et al., 2019). These limitations and concerns have spurred interest in actively developing label-free optical imaging technologies.

Terahertz (THz) radiation refers to the frequency band of 0.1–10 THz in the electromagnetic spectrum, corresponding to the wavelength of 3 mm–30 μm (Zhang et al., 2018). Due to the high sensitivity of THz spectral imaging technology to biomacromolecule, for example, proteins and nucleic acids exhibit unique vibration, rotation and conformational responses to THz radiation (Mittleman, 2018). Therefore, rich physical and chemical information inside biological samples can be provided in a label-free, non-invasive and non-ionizing manner (Chen et al., 2020; Zhou et al., 2021). At present, significant progress has been made in imaging biological samples using THz imaging technology (Wan et al., 2020; Chen et al., 2021; Ke et al., 2021). For example, THz imaging has successfully distinguished burnt skin tissue from normal skin tissue, and cancerous brain tissue from normal brain tissue (Bowman et al., 2017; Geng et al., 2019; Wu et al., 2019). Furthermore, the dehydration state of a single plant cell (~100 μm) was monitored and characterized by the THz near-field imaging technology based on the microstructure photo-conductive antenna (PCA; Li et al., 2020, 2022). Unfortunately, due to the inherent structural limitations of the PCA antenna micro-probes, it is difficult to further improve the resolution to the sub-micron or even nanometer level for the detection of a single organelle (~1 μm). At present, THz scattering-type scanning near-field optical microscope (THz s-SNOM) has the highest resolution in the field of THz imaging and is most likely to achieve a fine description of bacteria without damage. THz s-SNOM is an innovative ultra-high resolution optical microscope and spectral system based on atomic force microscopy (AFM) technology. The THz s-SNOM system relies on light scattering from metallized probes of nanoscale AFM to achieve near-field optical imaging and spectroscopy at subwavelength spatial resolution, independent of the wavelength of light. This method has been widely used to characterize nanoscale semiconductor devices (Yao et al., 2019), and free carrier concentration (Huber et al., 2008). A recent report has also shown that a single protein (~10 nm) can be imaged using a graphene-based THz imaging technique based on the scattering principle of nano-metal tips (Yang et al., 2021). Therefore, there is great potential to study single bacteria using the THz s-SNOM system.

This paper develops a new method for studying single bacteria using THz s-SNOM. Through the coupling effect of nanoscale radius of platinum probe and gold base, greatly enhanced THz near-field signal of biological samples, combined with the image processing method to remove the background noise and performing segmentation to obtain the subject target, has successfully demonstrated the morphological structure of individual bacteria.

## 2. Materials and methods

### 2.1. Sample preparation

The bacterial strains used in this study were Gram-negative *Escherichia coli* (*E. coli*, DH5α) and Gram-positive *Staphylococcus aureus* (*S. aureus*, ATCC 29213), both were from China Industrial Microbial Species Conservation Management Center. First, the strains were inoculated on LB medium (10 g/L tryptone, 5 g/L yeast extract and 10 g/L NaCl mixture), incubated overnight at 37°C for 12 h with constant temperature shaking, reached the growth stability stage, centrifuged at 8000 rpm for 15 min, and separated precipitates. The sediment was washed 3 times with 10 mmol/L, pH = 7.4 phosphoric acid buffer solution, and then re-suspended in PBS. A small amount of bacterial suspension was removed and diluted with ultra-pure water with a resistivity of 18.2 MΩ/cm for multiple times, then dropped onto the quartz sheet covered with nano-metal film using a 15 μl pipette, a drop of liquid is about 15 μl. Let stand for 24 h in a drying cabinet for complete drying, and then placed in the sample rack inside the system and fixed.

### 2.2. Experimental setup

**Figure 1:**
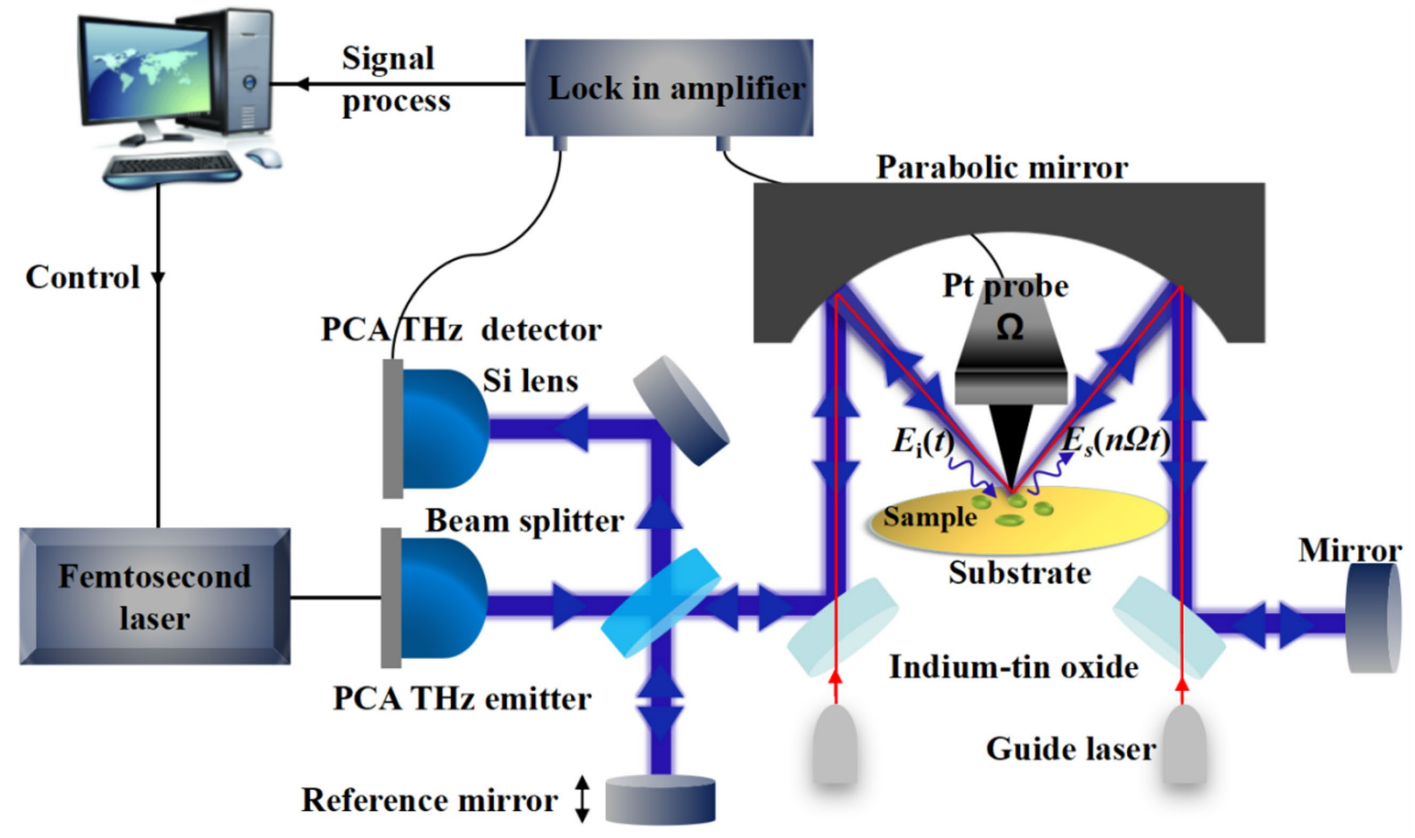
Schematic illustration of the THz s-SNOM setup and its use for single bacteria imaging.

The setup of the THz s-SNOM system produced by Neaspec is shown schematically in Figure 1. Ultrafast pulses produced by the femtosecond laser with a central wavelength of 1,550 nm are incident onto the photoconductive antenna (PCA) to excite the THz pulses. The input THz pulse is directed through a beam splitter and a reflector, and then focused onto the Pt probe by a special parabolic mirror which can support dual-beam operation. In order to maximize the focus of the invisible THz light on the tip of the needle, a set of guide light is introduced for collimation and beam steering. In this way, the local THz electric field can be significantly enhanced at the tip apex due to the lighting-rod effect and the dipole electric field enhancement effect (Raschke and Lienau, 2003; Keilmann and Hillenbrand, 2004). In order to enhance the THz near-field signal compared to background signal, the probe is controlled by an atomic force microscope (AFM) to interact with the sample in intermittent contact (tapping) mode. The scattering THz near-field signal is modulated by the probe oscillating at frequency *Ω* and collected by the parabolic mirror. Further, it is collimated and transmitted to the PCA detector by the interference detection scheme using a Michelson interferometer. The THz near-field amplitude and phase signal corresponding to the dielectric properties of the investigated sample can be acquired by using a lock-in amplifier at *nΩ* (*n* = 1, 2, 3…) for signal demodulation. In the process of imaging, the sample is scanned by the probe controlled by the AFM, and the near-field signals of each spatial point are obtained, which can be used for THz image reconstruction. In addition, the surface topography of the sample can be obtained simultaneously.

Due to the weak dielectric response of biological samples, the generated THz scattering near-field signals are weak. Our team have done a series of simulations to explore the enhancement effect of the dielectric effect of the substrate on the electric field at the tip. The simulation results show that for materials with obvious dielectric response, such as metals, the electric field at the tip will have a very large magnification, while when the substrate is semiconductor or other non-metallic materials with insignificant dielectric response, the electric field enhancement will be greatly reduced. The simulation pointed to the advantage of the very obvious difference between metal and nonmetal response to improve the contrast of the sample and substrate. In this way, we put the biological sample on the metal substrate made by micro-nano processing technology, resulting in obvious contrast between the two parts and improving the ability of sample recognition and image quality. The specific research content can be referred in Wang et al. (2020) and Dai et al. (2021). In this paper, vacuum electron beam evaporation coating was used to manufacture the gold-plated quartz slides as substrate. First, a 20 nm thick chromium film was plated on a 1 mm thick quartz sheet with a diameter of 5 cm, and then a 200 nm thick gold film was plated on the chromium film. In addition, the related parameters of the probe, such as material, length and curvature radius, also affect the strength of the THz scattering near-field signal. Based on the previous simulation and experimental results, a platinum probe with an ultra-long axis (~300 μm), tip curvature radius of about 50 nm, controlled by an AFM system, and vertical oscillation frequency of 25.1 kHz was selected.

### 2.3. Image processing

Since the THz near-field image is easily affected by noise, artifacts or inappropriate acquisition conditions, it is difficult to accurately obtain clear target samples. In this work, digital restoration methods are employed to process the original THz near-field amplitude and phase image.

#### 2.3.1. Dual-core gaussian filtering

In this paper, a dual kernel Gaussian filter is proposed to denoise the original image. Considering the two dimensional information of image pixel value range and spatial location, the dual-core Gaussian filter is designed, which can effectively improve the image signal-to-noise ratio (SNR) and solve the problem of image blur. The basic idea is to measure the similarity in pixel range and their spatial locations at the same time in the process of image denoising (Sugimoto and Kamata, 2015). Pixels with close spatial distance and small difference in pixel value are given larger weight, while pixels with far spatial distance and large difference in pixel value are given smaller weight. Its intuitionistic effect is reflected in the low contribution degree of pixels with longer spatial distance and larger pixel difference, which is conducive to the suppression of noise interference. The pixels with close spatial distance and small difference in pixel value contribute more, which is conducive to preserving image details, ultimately improving image SNR, reducing blur effect, and improving image quality.

The specific process is as follows: two pixels *I* (*x*_1_, *y*_1_) and *I* (*x*_2_, *y*_2_) in the original image are measured for similarity in value range and space by the Gaussian kernel function, and the formula is as follows:

$g\left(x,y\right)=\frac{1}{2\pi \sigma_{1}^{2}}e^{-\frac{\left||I\left(x_{1},y_{1}\right)+I{\left(x_{2},y_{2}\right)}^{2}|\right|}{2\sigma_{1}^{2}}}$ (1)

$f\left(x,y\right)=\frac{1}{2\pi \sigma_{2}^{2}}e^{-\frac{{\left(x_{1}-x_{2}\right)}^{2}+{\left(y_{1}-y_{2}\right)}^{2}}{2\sigma_{2}^{2}}}\;$ (2)

where g(x,y) is the value range similarity measure, f(x,y) is the spatial similarity measure, σ _1_ represents the Gaussian distribution variance of pixel value level similarity, σ _2_ represents the Gaussian distribution variance of distance measure. The coefficients representing the range and spatial similarity measure are multiplied and used to estimate the pixel weight coefficient, namely.

$w\left(x,y\right)=g\left(x,y\right).f\left(x,y\right)$ (3)

Then the original image *I* (*x*, *y*) after dual-core Gaussian filtering is:

$I^{’}\left(x,y\right)=\frac{\sum w\left(x,y\right).I\left(x,y\right)}{\sum w\left(x,y\right)}$ (4)

The key parameters to realize dual-core Gaussian filtering are the selection of sigmaColor and sigmaSpace. The values of sigma are larger, the edge detail information is more fuzzy. But the values should not be too small, otherwise there are no filtering effect. On balance, we tested a good range between 180 and 200.

#### 2.3.2. Grabcut segmentation based on SLIC

In this paper, simple linear iterative clustering is used to preprocess the image, and the adjacent pixels with similar color are divided into a block, which is called a super pixel. Then Grabcut algorithm is used to process the super pixel image, which greatly improves the efficiency of the algorithm. Simple linear iterative clustering (SLIC) algorithm can dynamically adjust the appropriate parameter values according to the texture complexity of different regions of the image, which is conducive to reduce the impact of the difference of super pixel shape on the segmentation effect in subsequent processing (He et al., 2022). Then the GmbCut algorithm is used to segment the image on the foreground/background area of the super pixel map. Gaussian mixture model (GMM) is mainly used for iterative image processing. Each iteration is based on the GMM parameters generated by the segmentation results of the previous iteration to reconstruct the graph model and calculate the segmentation process.

## 3. Results and discussion

**Figure 2:**
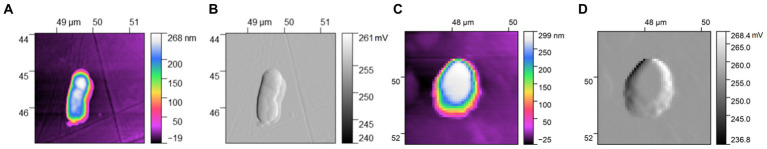
AFM images of single bacterium: **(A)** topographic map of *Escherichia coli*; **(B)** amplitude image of *E. coli*; **(C)** topographic map of *Staphylococcus aureus*; **(D)** amplitude image of *S. aureus*.

Figure 2 is the result of AFM imaging of single *E. coli* and *S. aureus*, where Figures 2A,C are topographic maps, which clearly show the morphological information of single bacterium. Figures 2B,D are amplitude images, which describe the surface topography of the bacteria. A rod form of the bacterium can be clearly observed in Figures 2A,B, it is bluntly rounded at both ends and has flagella at the tail, which is clear that *E. coli* has been detected. The single *E. coli* is about 1.7 μm long and 0.5 μm wide. Meanwhile, it becomes higher from the edge to the middle, and the highest point is about 280 nm. Figures 2C,D depict a spherical bacterium, which is approximately 1.8 μm in diameter and 300 nm at its highest. It is clearly *S. aureus*. The results in this paper are in good agreement with the previous results of bacterial morphology of *E. coli* and *S. aureus*, and the specific results can be referred to the book (Ghalem, 2015) and reference (Klainer and Perkins, 1970).

**Figure 3:**
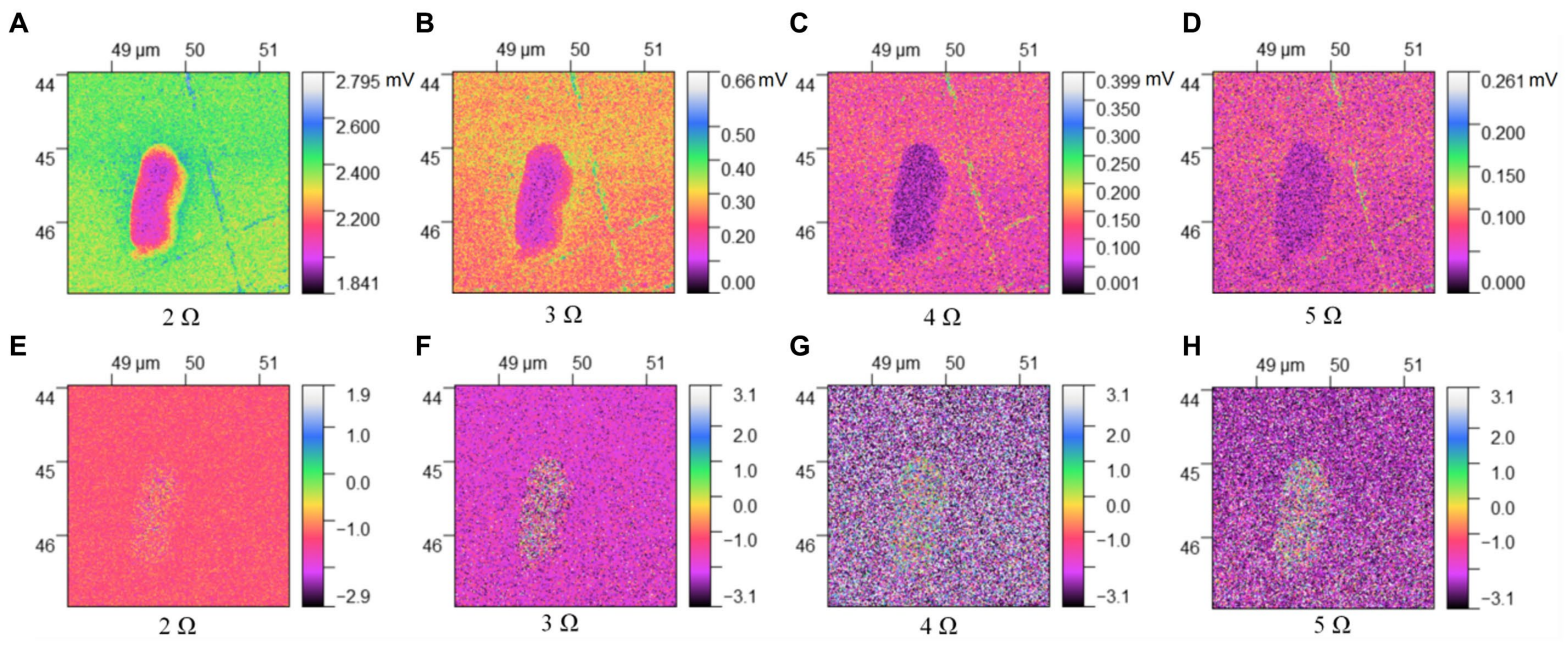
THz near-field imaging of *E. coli*: **(A–D)** amplitude image; **(E–H)** phase image.

**Figure 4:**
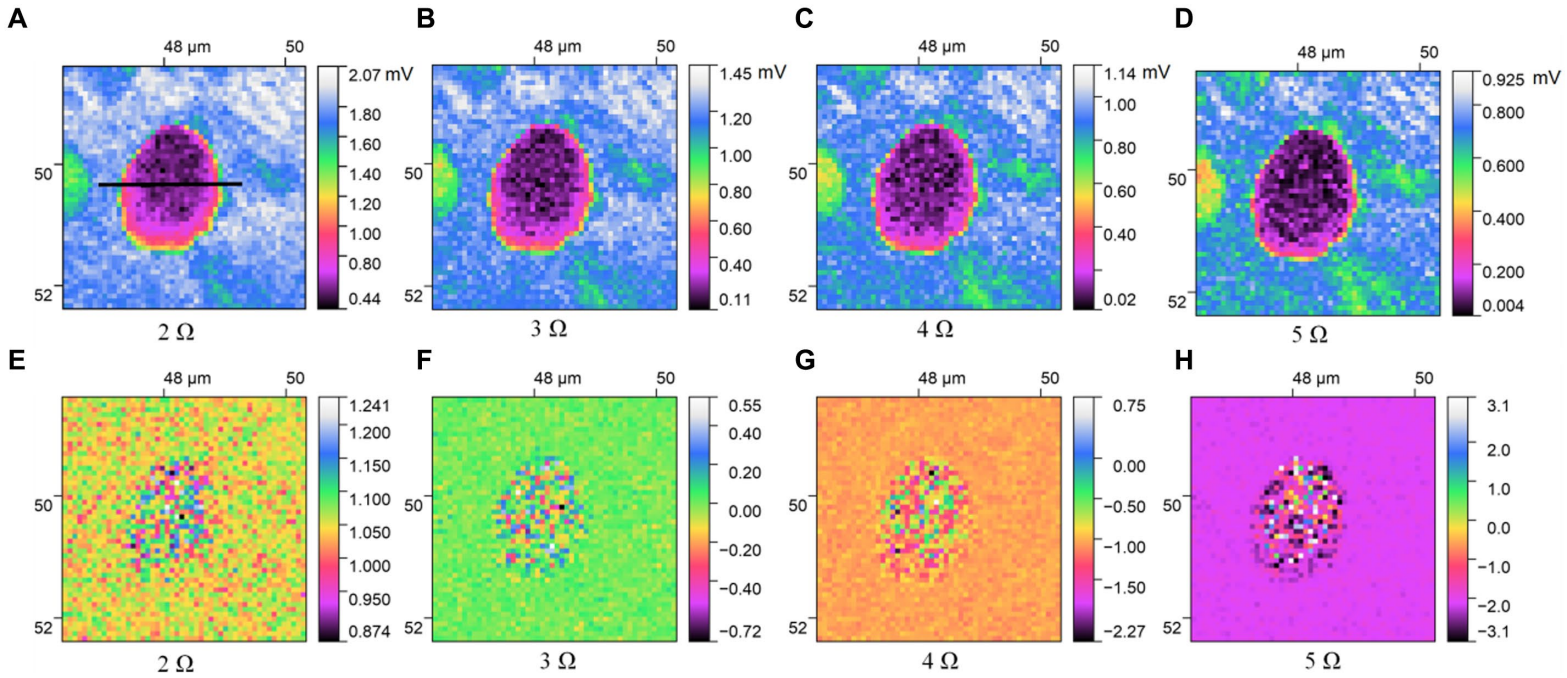
THz near-field imaging of *S. aureus*: **(A–D)** amplitude image; **(E–H)** phase image.

**Figure 5:**
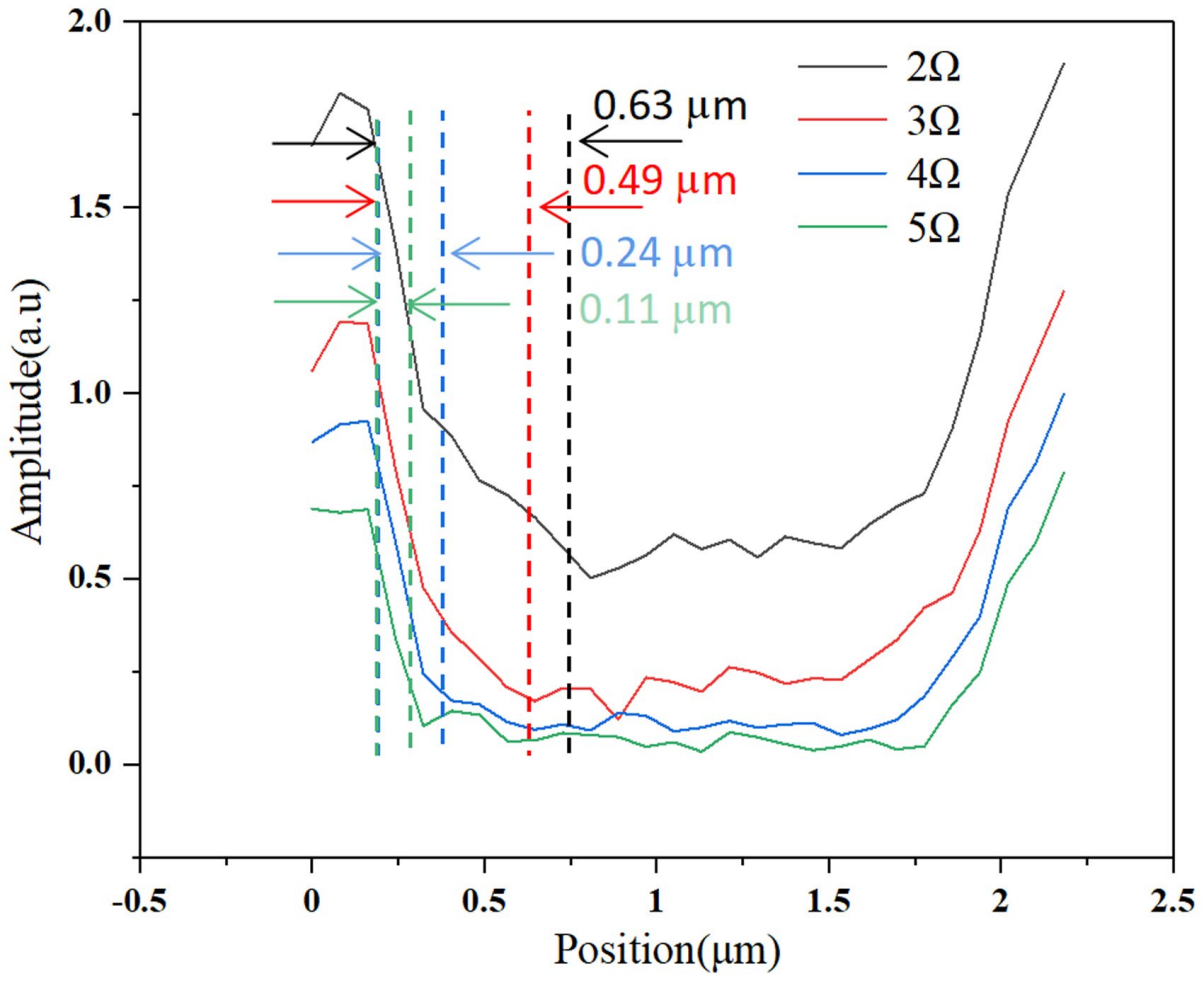
Spatial resolution of THz near-field imaging of the bacterium *E. coli*. Near-field signal amplitude variation along the black line shown in Figure 4A, for various harmonic orders of signal demodulation. Spatial resolution is estimated based on the spatial spread of the point where the signal is 90% of its maximum to the point where the signal is 10% of its maximum.

Figures 3, 4 describe the two different bacteria by using THz near-field imaging, where (A–D) and (E–H) are THz amplitude and phase images at different harmonic demodulation (from 2 Ω to 5 Ω), respectively. Refer to Figure 2, THz near-field amplitude and phase imaging of the bacterium are consistent with topographic maps, and both can accurately describe the shape and size of the individual bacterium. From the results shown in Figures 3, 4, a clear regional division between the biological samples and the substrate can be easily identified based on the THz near field images. It is generally believed that the resolution of s-SNOM is closely related to the harmonic frequency of signal extraction (Mastel et al., 2018; Dai et al., 2019). To test the detailed resolution of biological samples at different harmonics, we select a line on the amplitude image that crosses the boundary between the sample and the substrate (the black line in Figure 4A) to derive the resolution, along which signal values are obtained as a function of position. These are shown in Figure 5, for the harmonics demodulation frequencies from 2 Ω to 5 Ω. In Figure 5, according to the 10–90% rule, the spatial resolution of the near-field image demodulated at the fifth harmonic of the tip modulation frequency is obtained: corresponding to 2 Ω to 5 Ω, the resolution is 0.63 μm, 0.49 μm, 0.24 μm and 0.11 μm, respectively. Our experimental results are consistent with reported findings previously, that is, the resolution of higher harmonics is better than that of lower harmonics (Fikri et al., 2004). On the other hand, as the order increases, the demodulated phase image showing the outline of the bacteria becomes clearer, which can be seen in Figures 3E–H, 4E–H. In our example, the resolution obtained at 5 Ω is the best of all harmonics. Obviously, if we need to obtain sample information at the highest possible resolution, the demodulation signal at 5 Ω would be a good choice. In addition, we found that the intensity of the near-field signal decreased sharply as the demodulation frequency increased from 2 Ω to 3 Ω, which can be attributed to the high frequency attenuation suffered by the demodulation signal during the lock-in process (Wurtz et al., 1999; Mastel et al., 2018). Except for the almost invisible differences, all four amplitude images have good qualities and describe morphology of bacteria clearly, which also indicates that signal strength alone does not always make better image quality, and the intensity contrast between biological samples and substrate is also a key factor in evaluating the quality of near-field signals (Dai et al., 2021).

**Figure 6:**
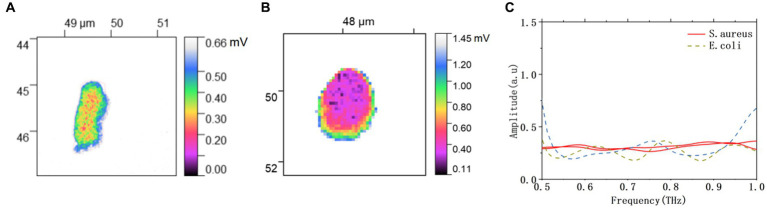
Processed THz near-field imaging of **(A)** *E. coli*, and **(B)** *S. aureus*. **(C)** THz near-field spectral signals of *E. coli* and *S. aureus*.

In this study, we have demonstrated that THz spectral images can describe the morphology of bacteria as well as the AFM images. More importantly, THz waves can penetrate into the bacteria, and demonstrate the distribution of substances inside the bacteria according to the differences in the spatial variation of composition and dielectric properties interior of the bacteria. The THz spectral images of bacterial morphology have been denoised and segmented by the image processing method presented in Section 3.1, as shown in Figure 6. In addition to the morphology of bacteria, the internal structure of bacteria can also be observed. In particular, pseudonuclei made up of loosely coiled circular DNA molecules can be observed in *E. coli* (Figure 6A), and the structure of *E. coli* can be referred to the literature (Verma et al., 2019). By counting the number of pixels in the bacterial regions, the calculated areas of *E. coli* and *S. aureus* were about 0.747 μm^2^ and 1.296 μm^2^, respectively. As shown in Figure 6C, the THz near-field signals on bacterial bodies at different positions have been extracted from the THz spectra image in Figures 6A,B. The near-field signals of *E. coli* and *S. aureus* are obviously different. The near field signals of *E. coli* are more stable, while that of *S. aureus* fluctuate with increasing frequency. The two types of bacteria can also be easily identified by differences in their THz spectral information.

## 4. Conclusion

In this paper, an advanced THz super-resolution imaging method is proposed. Specifically, the THz near-field signal is enhanced by the coupling effect between the probe and the gold film substrate, and the THz spectral image of a single bacterium is successfully obtained by using the THz s-SNOM system. Further, combining with image processing method to remove background noise and obtain biological image segmentation, we were able to clearly show the internal nucleoid structure of *E. coli*. The method presented in this paper has been applied to the monomer identification of *E. coli* and *S. aureus*. Super-resolution of THz morphological imaging and THz spectral differences can be used to distinguish the two monomers easily. Hence, this method paves a new avenue for the label-free study of individual bacteria.

## Data availability statement

The original contributions presented in the study are included in the article/supplementary material, further inquiries can be directed to the corresponding author.

## Author contributions

All authors listed have made a substantial, direct, and intellectual contribution to the work, and approved it for publication.

## Funding

This work is supported by the Major Instrumentation Development Program of the Chinese Academy of Sciences of China (ZDKYYQ20220008), the National Natural Science Foundation of China (NSFC) under grants 61875051 and 62271439, and the Natural Science Foundation of Zhejiang Province (ZJNSF) under grant LR21F010002.

## Conflict of interest

The authors declare that the research was conducted in the absence of any commercial or financial relationships that could be construed as a potential conflict of interest.

## Publisher’s note

All claims expressed in this article are solely those of the authors and do not necessarily represent those of their affiliated organizations, or those of the publisher, the editors and the reviewers. Any product that may be evaluated in this article, or claim that may be made by its manufacturer, is not guaranteed or endorsed by the publisher.

## References

[ref1] Bowman T. Wu Y. Gauch J. Campbell L. K. el-Shenawee M. (2017). Terahertz imaging of three-dimensional dehydrated breast cancer tumors. J. Infrared Millim. Terahertz Waves. 38, 766–786. doi: 10.1007/s10762-017-0377-y

[ref2] Chen X. Y. Tian Z. Lu Y. C.. (2020). Electrically tunable perfect terahertz absorber based on a Graphene Salisbury screen hybrid metasurface. Adv. Opt. Mater. 8:1900660. doi: 10.1002/adom.201900660

[ref3] Chen A. Virk A. Harris Z. Abazari A. Honkanen R. Arbab M. H. (2021). Non-contact terahertz spectroscopic measurement of the intraocular pressure through corneal hydration mapping. Biomed. Opt. Express 12, 3438–3449. doi: 10.1364/BOE.423741, PMID: 34221670 PMC8221940

[ref4] Cosentino M. Canale C. Bianchini P. Diaspro A. (2019). AFM-STED correlative nanoscopy reveals a dark side in fluorescence microscopy imaging. Sci. Adv. 5:eaav8062. doi: 10.1126/sciadv.aav8062, PMID: 31223651 PMC6584704

[ref5] Dai G. Geng G. Zhang X. Wang J. Chang T. Cui H. L. (2019). W-band near-field microscope. IEEE Access. 7, 48060–48067. doi: 10.1109/ACCESS.2019.2907742

[ref6] Dai G. B. Wang J. Zhang X. X.. (2021). Low terahertz-band scanning near-field microscope with 155-nm resolution. Ultramicroscopy 226:113295. doi: 10.1016/j.ultramic.2021.113295, PMID: 34000640

[ref7] Fikri R. Grosges T. Barchiesi D. (2004). Apertureless scanning near-field optical microscopy: numerical modeling of the lock-in detection. Opt. Commun. 232, 15–23. doi: 10.1016/j.optcom.2003.12.027

[ref8] Gahlmann A. Moerner W. (2014). Exploring bacterial cell biology with single-molecule tracking and super-resolution imaging. Nat. Rev. Microbiol. 12, 9–22. doi: 10.1038/nrmicro3154, PMID: 24336182 PMC3934628

[ref9] Geng G. Dai G. Li D. Zhou S. Li Z. Yang Z.. (2019). Imaging brain tissue slices with terahertz near-field microscopy. Biotechnol. Prog. 35:e2741. doi: 10.1002/btpr.2741, PMID: 30414311

[ref10] Ghalem B. R. (2015). “*Escherichia coli* and *Staphylococcus aureus* most common source of infection,” in The Battle Against Microbial Pathogens: Basic.

[ref11] He F. Y. Mahmud M. A. P. Kouzani A. Z.. (2022). An improved SLIC algorithm for segmentation of microscopic cell images. Biomed. Signal Process. Control. 73:103464. doi: 10.1016/j.bspc.2021.103464

[ref12] Huber A. J. Keilmann F. Wittborn J. Aizpurua J. Hillenbrand R. (2008). Terahertz near-field nanoscopy of mobile carriers in single semiconductor nanodevices. Nano Lett. 8, 3766–3770. doi: 10.1021/nl802086x, PMID: 18837565

[ref13] Karbelkar A. A. Furst A. L. (2020). Electrochemical diagnostics for bacterial infectious diseases. ACS Infect. Dis. 6, 1567–1571. doi: 10.1021/acsinfecdis.0c00342 32646219

[ref14] Ke L. Wu Q. Y. S. Zhang N. Yang Z. Teo E. P. W. Mehta J. S.. (2021). Terahertz spectroscopy analysis of human corneal sublayers. J. Biomed. Opt. 26:043011. doi: 10.1117/1.JBO.26.4.043011, PMID: 33899380 PMC8071781

[ref15] Keilmann F. Hillenbrand R. (2004). Near-field microscopy by elastic light scattering from a tip. Philos. Trans. R. Soc. A Math. Phys. Eng. Sci. 362, 787–805. doi: 10.1098/rsta.2003.1347, PMID: 15306494

[ref16] Klainer A. S. Perkins R. L. (1970). Antibiotic-induced alterations in the surface morphology of bacterial cells: a scanning-beam electron miscroscopy study. J. Infect. Dis. 122, 323–328. doi: 10.1093/infdis/122.4.323 4994168

[ref17] Li Z. Yan S. Zang Z. Geng G. Yang Z. Li J.. (2020). Single cell imaging with near-field terahertz scanning microscopy. Cell Prolif. 53:e12788. doi: 10.1111/cpr.12788, PMID: 32153074 PMC7162806

[ref18] Li Z. Zang Z. Wang J. Lu X. Yang Z. Wang H.. (2022). In situ cell detection using terahertz near-field microscopy. IEEE T. THz. Sci. Techn. 12, 457–463. doi: 10.1109/TTHZ.2022.3170010

[ref19] Lucidi M. Tranca D. E. Nichele L. Ünay D. Stanciu G. A. Visca P.. (2014). A collection of scattering-type scanning near-field optical microscopy and atomic force microscopy images of bacterial cells. GigaSciencen. 9:giaa129. doi: 10.1093/gigascience/giaa129, PMID: 33231675 PMC7684706

[ref20] Mastel S. Govyadinov A. A. Maissen C. Chuvilin A. Berger A. Hillenbrand R. (2018). Understanding the image contrast of material boundaries in IR nanoscopy reaching 5nm spatial resolution. ACS Photonics. 5, 3372–3378. doi: 10.1021/acsphotonics.8b00636

[ref21] Mittleman D. M. (2018). Twenty years of terahertz imaging. Opt. Express 26, 9417–9432. doi: 10.1364/OE.26.009417 29715894

[ref22] Raschke M. B. Lienau C. (2003). Apertureless near-field optical microscopy: tip–sample coupling in elastic light scattering. Appl. Phys. Lett. 83, 5089–5091. doi: 10.1063/1.1632023

[ref23] Rust M. J. Bates M. Zhuang X. W. (2006). Sub-diffraction-limitimaging by stochastic optical reconstruction microscopy (STORM). Nat. Methods 3, 793–796. doi: 10.1038/nmeth929, PMID: 16896339 PMC2700296

[ref24] Sugimoto K. Kamata S. I. (2015). Efficient constant-time gaussian filtering with sliding dct/dst-5 and dual-domain error minimization. ITE Trans. Media Technol. Appl. 3, 12–21. doi: 10.3169/mta.3.12

[ref25] Verma S. C. Zhong Q. Adhya S. L. (2019). Architecture of the *Escherichia coli* nucleoid. PLoS Genet. 15:e1008456. doi: 10.1371/journal.pgen.1008456, PMID: 31830036 PMC6907758

[ref26] Wan M. Healy J. J. Sheridan J. T. (2020). Terahertz phase imaging and biomedical applications - sciencedirect. Opt. Laser Technol. 122:105859. doi: 10.1016/j.optlastec.2019.105859

[ref27] Wang J. Yan S. H. Li Z. Zang Z. Lu X. Cui H. L. (2020). Single-cell terahertz spectral characteristics in simulated scattering near-field imaging mode. OSA Continuum. 3, 2096–2105. doi: 10.1364/OSAC.400827

[ref28] Wu L. Xu D. Wang Y. Liao B. Yao J. (2019). Study of in vivo brain glioma in a mouse model using continuous-wave terahertz reflection imaging. Biomed. Opt. Express 10, 3953–3962. doi: 10.1364/BOE.10.003953, PMID: 31452987 PMC6701535

[ref29] Wurtz G. Bachelot R. Royer P. (1999). Imaging a gaalas laser diode in operation using apertureless scanning near-field optical microscopy. Eur. Phys. J. Appl. Phys. 5, 269–275. doi: 10.1051/epjap:1999139

[ref30] Yang Z. Tang D. Hu J. Tang M. Zhang M. Cui H. L.. (2021). Near-field nanoscopic terahertz imaging of single proteins. Small 17:2005814. doi: 10.1002/smll.202005814, PMID: 33306275

[ref31] Yao Z. Semenenko V. Zhang J. Mills S. Liu M. (2019). Photo-induced terahertz near-field dynamics of graphene/inas heterostructures. Opt. Express 27, 13611–13623. doi: 10.1364/OE.27.013611, PMID: 31163822

[ref32] Zhang J. Chen X. Mills S. Ciavatti T. Yao Z. Mescall R.. (2018). Terahertz nanoimaging of graphene. ACS Photonics. 5, 2645–2651. doi: 10.1021/acsphotonics.8b00190

[ref33] Zhou R. Wang C. Huang Y. Huang K. Wang Y. Xu W.. (2021). Label-free terahertz microfluidic biosensor for sensitive DNA detection using graphene-metasurface hybrid structures. Biosens. Bioelectron. 188:113336. doi: 10.1016/j.bios.2021.113336, PMID: 34022719

